# Effect of fenestrated sterile drape and face mask on bacterial dispersion toward the periocular area during intravitreal injection

**DOI:** 10.1038/s41598-023-37091-3

**Published:** 2023-06-19

**Authors:** Nopasak Phasukkijwatana, Rawi Jongpipatchai, Peerawoot Phuksapaisalsilp, Sujiraphong Pharkjaksu, Popchai Ngamskulrungroj, Supalert Prakhunhungsit

**Affiliations:** 1 Department of Ophthalmology, Faculty of Medicine Siriraj Hospital, Mahidol University, 2 Wanglang Road, Bangkoknoi, Bangkok, 10700 Thailand; 2 Department of Microbiology, Faculty of Medicine Siriraj Hospital, Mahidol University, 2 Wanglang Road, Bangkoknoi, Bangkok, 10700 Thailand

**Keywords:** Macular degeneration, Retinal diseases, Bacterial infection

## Abstract

This experimental crossover study was performed to investigate whether fenestrated surgical drapes (covering the nose and mouth but with an opening over the periorbital area) with or without patients’ surgical face masks increase periorbital bacterial dispersion during simulated intravitreal injection conditions. Each of the 16 healthy volunteers performed 14 scenarios involving different mask and drape conditions in both silent and speaking situations. In each scenario, the subject lay down flat on the back with a blood agar plate being held at the inferior orbital rim perpendicular to the face to capture airflow from breathing/speaking. Another blood agar plate placed 50 cm away from the subject served as an experimental control. A total of 224 experiments were performed. Speaking situations significantly showed more colony forming units (CFUs) compared with their controls (P = 0.014). There were no significant differences in CFUs between wearing vs not wearing the masks (P = 0.887 for speaking and P = 0.219 for silent) and using vs not using the drapes (P = 0.941 for speaking and P = 0.687 for silent). Reusable and disposable drapes were also not significantly different (P = 1.00 for speaking and P = 0.625 for silent). *Streptococcus* spp., the oropharyngeal microbiota, were only cultivated from speaking scenarios. While refraining from speaking (for both practitioners and patients) is the mainstay of reducing bacterial dispersion and risks of post-injection endophthalmitis, the use of fenestrated surgical drapes or patients’ face masks did not significantly affect the amount of bacterial dispersion toward the periorbital area.

## Introduction

Intravitreal injections of medications, particularly anti-vascular endothelial growth factors (anti-VEGF), are currently considered as one of the most common procedures in medicine to treat diseases such as neovascular age-related macular degeneration and diabetic macular edema^1^. In general, the treatment has minimal side effects and good safety profiles. However, post-injection endophthalmitis is always a major concern due to its devastating visual outcome^2^. Although the incidence of endophthalmitis post intravitreal treatment was reported to vary from 0.004 to 0.036%^3,4^, great efforts have been employed to reduce the risk of infection as much as possible. Number of studies evaluated potential risk factors of intravitreal injection-related endophthalmitis and oropharyngeal droplet transmission was one of the major risk factors^2,5–7^. Previous studies suggested that *Streptococcus* spp., one of the respiratory tract microbes, was associated with poor endophthalmitis outcomes^6,8^. For these reasons, several protocols such as ‘no talking’ and ‘physicians wearing face mask’ policies were recommended^5,9,10^.

During the COVID-19 pandemic, face mask wearing has become a general routine to decrease the risk of coronavirus infection. However, there was a concern that face masks used by patients may increase oropharyngeal bacterial dispersion toward the eyes due to upward air flow from breathing or speaking^11,12^. Several experiments suggested that taping of superior rim of the masks or using N95 face masks could reduce the upward air flow and bacterial dispersion^11,13–15^.

In many ophthalmology centers including our center at Siriraj hospital, sterile fenestrated surgical drapes (with an opening over the periorbital area) have been employed to cover patients’ faces before giving intravitreal injections to keep the area sterile and to increase awareness of serious procedures for both physicians and patients, although there was no significant evidence that the routine use of the drapes could reduce endophthalmitis rate^16,17^. On the other hand, similar to face masks, a fenestrated surgical drape covering the nose and mouth but with an opening over the periorbital area can potentially increase air flow and pharyngeal bacterial dispersion toward the periorbital area during intravitreal injection procedures. Moreover, in COVID-19 era, combining effects of patients’ face masks and fenestrated surgical drapes on the bacterial dispersion are unknown.

Thus, this study aims to investigate the amount of bacterial dispersion toward the periorbital area associated with the use of fenestrated surgical drapes with or without patients’ face masks during simulated intravitreal injection situations.

## Methods

### Subjects

This experimental multiple crossover study was approved by the Siriraj Institutional Review Board, COA No. Si508/2021, and was performed in accordance with the Declaration of Helsinki. The study was registered in the Thai Clinical Trials Registry, No. TCTR20221222002 (22/12/2022). Healthy volunteers aged 18–80 years old were included. Subjects with the following criteria were excluded: fever or respiratory symptoms or systemic antibiotics administration within the past 2 weeks, chronic airway diseases, facial skin or ocular infection, inability to lie down flat on the back and allergy to adhesive paper tape (3M™ Micropore Tape 1530-1). Informed consent was obtained from all participants.

### Study procedures

The study was conducted in a closed air-conditioned room, used for everyday intravitreal injections at the Intravitreal Injection Clinic, Siriraj Hospital. The room was disinfected with UVC light (Philips UVC disinfection desk lamp 24W S TC) for 30 min before each subject. There were no specialized air filtration equipment or positive/negative pressure ventilation systems.

In each experiment scenario, a subject was instructed to lie down flat on an injection bed. An investigator stood behind the head position of the bed while holding a blood agar plate for 2 min at the inferior orbital rim perpendicular to the face of the subject. The second investigator held a second blood agar plate, serving as an experiment control, 50 cm away from the subject, at the same height and in a similar orientation to the first plate. Both investigators wore N95 mask (3M™ Aura™ 1870+) and remained silent throughout the experiment. Prior to the experiment phase in each subject, a blood agar plate was placed at the head position of the bed for 2 min without people in the room to serve as a room condition control. All blood agar plates were sent for bacterial culture tests.

Each subject was instructed to perform 7 experiment scenarios of using fenestrated sterile drapes and/or surgical face masks. The scenarios were the following: (1) no fenestrated sterile drape and face mask; (2) face mask only; (3) face mask with taping of the superior rim of the mask with adhesive paper tape (3M™ Micropore Tape 1530-1); (4) reusable fenestrated sterile cloth drape without face mask; (5) reusable fenestrated sterile cloth drape with face mask; (6) disposable fenestrated sterile drape without face mask; and (7) disposable fenestrated sterile drape with face mask. For all subjects, the face masks were worn tight fit to the nose (with the malleable nose bridge metal strip firmly pressed around the nose) and the openings of the fenestrated sterile drapes were centered over the right eye. Each scenario was performed in both silent and speaking conditions (14 scenarios in total). For speaking conditions, the subject was instructed to count consecutive numbers (in the Thai language) out loud for 2 min. The surgical face masks used in this study were disposable 3-ply face masks with ear loops and commercially available under the tradename Medimask (ASTM, F2100, level 1). The reusable fenestrated sterile cloth drapes (45 × 45 cm) and the disposable fenestrated sterile drapes (35 × 45 cm, polyethylene nonwoven sheet, Thai Gauze Co., Ltd.) contained the oval apertures of 35 × 50 mm. For each participant, the same mask and drapes were used for all of the 14 scenarios.

To minimize carryover effects of each scenario to one another on the bacterial culture outcomes, the sequences of the scenarios were randomized for each subject prior to the experiments. The randomization first involved sequences of wearing/not wearing face masks and then sequences of covering/uncovering the faces with different types of sterile drapes. For each subject, the same randomized sequence was used for both silent and speaking conditions. Speaking scenarios were always performed after silent scenarios in every subject as speaking might cause more bacterial dispersion. There was a 2-min washout period in which the investigators all wearing N95 and remaining silent in the injection room between each scenario.

All blood agar plates were sealed, labeled by anonymous code number and transferred to the Microbiology Laboratory at Siriraj hospital where they were incubated for 72 h at 37 °C in a 5% carbon dioxide–rich environment. The number of bacterial colonies per plate were counted using standard laboratory techniques by microbiologists. The bacterial species were identified using the conventional biochemical techniques of the Department of Microbiology, Faculty of Medicine Siriraj Hospital (test code 201201 bacteria: culture [aerobic]). The microbiologists were blinded to the experiment scenarios.

### Statistical analysis

The amount of bacteria in colony forming units (CFUs) were compared between different scenarios using Wilcoxon signed-rank test. When the CFUs were either 0 or 1 in each subject, the comparisons were made using McNemar’s test.

The effect of each of the factors including: (1) the presence/absence of face masks; (2) the presence/absence of fenestrated sterile drapes; and (3) the types of drapes (reusable and disposable) was analysed. Different scenarios were grouped according to the above factors. In particular, the presence of face masks included scenarios 2, 5 and 7. The absence of face masks comprised scenarios 1, 4 and 6. The presence of fenestrated sterile drapes consisted of scenarios 4, 5, 6 and 7. The absence of fenestrated sterile drapes included scenarios 1 and 2. Reusable drape consisted of scenarios 4 and 5 and disposable drape was composed of scenarios 6 and 7. The bacterial CFUs were summed within group and compared between different groups.

### Conference presentation

The work has been presented at the 128th annual meeting of the Korean Ophthalmological Society, Seoul, Korea on 29th October, 2022.

## Results

**Figure 1:**
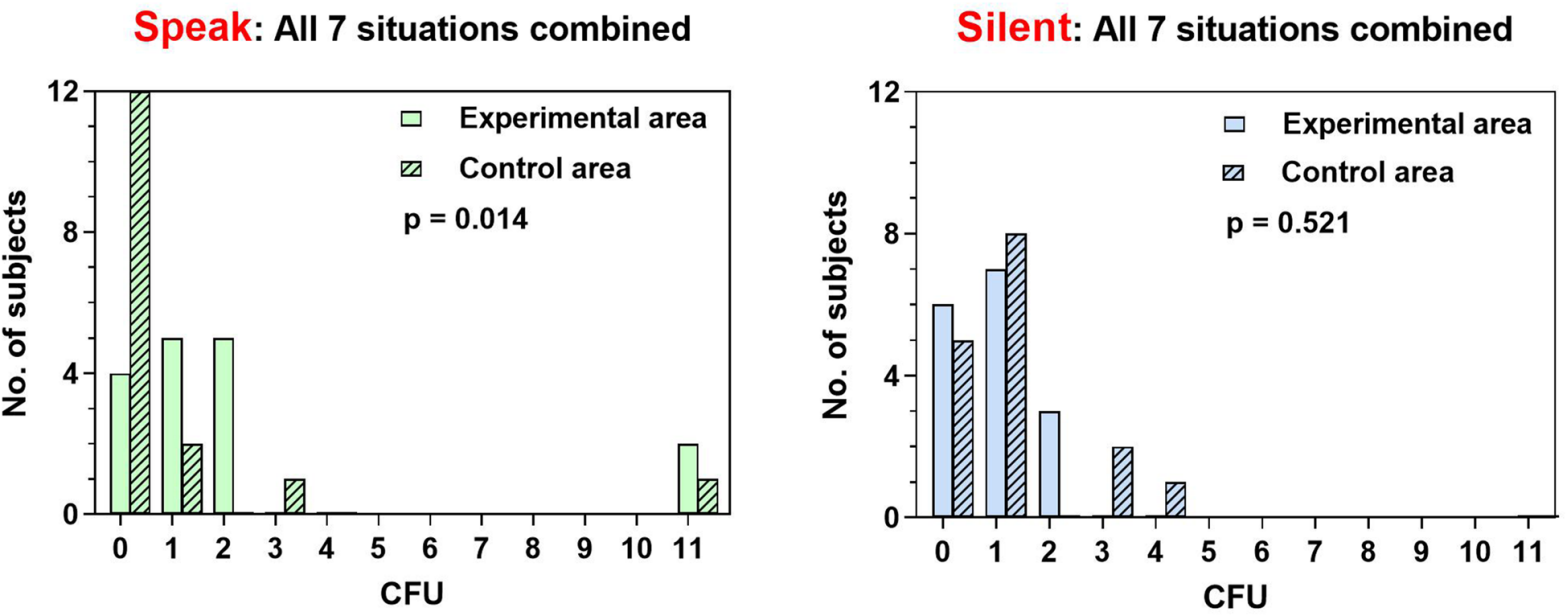
Comparisons of the amount of bacteria in colony forming unit (CFU) between experimental and control blood agar plates in speaking and silent scenarios.

Sixteen volunteers fulfilled the inclusion and exclusion criteria and were recruited into the study. The mean ± standard deviation of age was 30 ± 5.8 years old and 63% were female. The total number of blood agar was 464 plates from 224 experiments. All 16 room control agar plates did not grow any bacteria. The distributions of subjects according to the amount of bacteria in CFUs in all speaking scenarios and all silent scenarios were shown in Fig. 1. When all 7 scenarios were combined, speaking condition significantly showed more bacterial dispersion compared with their controls (P = 0.014). In contrast, the bacterial dispersion in silent scenarios was not statistically different than their controls (P = 0.521).

**Figure 2:**
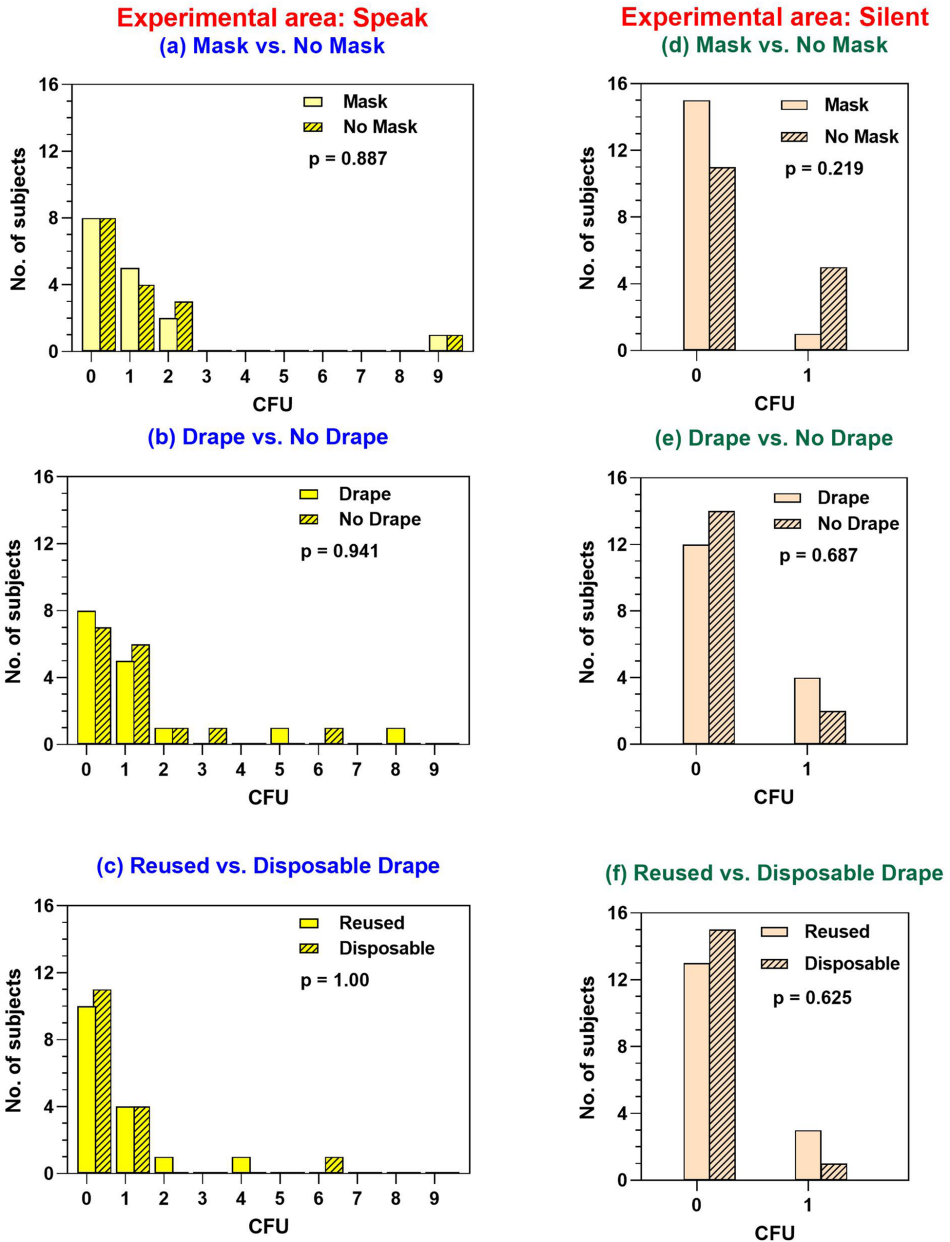
The distributions of subjects according to the amount of bacterial colony forming units comparing between wearing vs not wearing face masks, using vs not using fenestrated sterile drapes and using reusable vs disposable drapes in speaking and silent scenarios.

The effects of wearing face masks, using fenestrated sterile drapes, or types of drapes were separately analysed (Fig. 2). There were no significant differences in bacterial CFUs between wearing and not wearing face masks (P = 0.887 for speaking and P = 0.219 for silent conditions). No significant differences were found between using and not using fenestrated sterile drapes (P = 0.941 for speaking and P = 0.687 for silent). The reusable and disposable drapes were also not significantly different (P = 1.00 for speaking and P = 0.625 for silent). Moreover, taping of the superior edge of the face masks did not show significant differences in bacterial CFUs compared with face masks with no taping (P = 0.125 for speaking and P = 0.219 for silent conditions).

A total of 87 bacterial CFUs grew on the blood agar plates (53 in experiment plates and 34 in control plates). *Staphylococcus* spp. and *Micrococcus luteus* were the most common bacterial strains identified. They were found equally in both experiment and control plates (Table 1). *Streptococcus* spp., the oropharyngeal flora organisms, were cultivated only from speaking experiment scenarios.

**Table 1:** Microbial strains identified in this study.

Microbial strains	Colony forming units	Percentage (%)
Experiment plates		
* Staphyloccus* spp*.*	15	17.2
* Micrococcus luteus*	12	13.8
Streptococci-alpha hemolytic	6	6.9
* Coryneform bacteria*	5	5.7
* Brevundimonas diminuta*	3	3.4
* Moraxella* spp*.*	2	2.3
* Aerobic actinomycetes*	1	1.1
* Alloiococcus otitis*	1	1.1
* Kocuria kristinae*	1	1.1
Yeasts	1	1.1
Unidentified organisms	6	6.9
Control plates		
* Staphylococcus* spp*.*	17	19.5
* Micrococcus luteus*	11	12.6
Suspected mold	3	3.4
Coryneform bacteria	2	2.3
Unidentified organisms	1	1.1
Total	87	100

## Discussion

Due to the COVID-19 pandemic, it has become a general practice in many hospitals to wear face masks for both physicians and patients. Recent studies found increased air leaks from the superior rims of face masks^11,14,15^. This has raised concerns about increasing periorbital oropharyngeal bacterial dispersion from patients’ face masks and post-intravitreal injection endophthalmitis risk. However, there was still a lack of data regarding periorbital air leaks and bacterial dispersion relating to fenestrated surgical drapes. This study addressed this issue and demonstrated that using fenestrated surgical drapes, either reusable cloth drapes or disposable drapes, did not lead to more bacterial growth on the agar plates during simulated intravitreal injection conditions. The combination of using the drapes and properly worn patients’ surgical masks (tight-fitting) also did not affect the bacterial growth. However, speaking by patients was significantly associated with more periorbital bacterial dispersion.

According to our results, wearing tight-fitting surgical masks on patients showed no significant impact on periorbital bacterial growth compared with no masks in both silent and speaking conditions. Moreover, taping of the superior edge of the surgical masks to seal the gap between the masks and nasal bridges did not show significant differences in bacterial dispersion compared with no taping. Recently, Patel et al. found no significant differences in bacterial dispersion between wearing tight-fitting surgical masks and no masks similar to our study^13^. However, they did find significant reduction of periorbital bacterial dispersion by taping the top edge of the surgical masks compared with no taping. Different facial anatomy between Asian subjects in our study and Caucasian subjects in their study might partly explain the discordant result. Reavis et al. also did not find a significant reduction in bacterial dispersion by taping the superior aspect of the surgical masks^15^. Another study showed reduced air particle counts, indicating exhaled bacterial flora contamination, from the top of cloth masks in speaking condition by taping the top edges of the cloth masks. However, the air particle counts detected in the taping condition for surgical masks (not cloth masks) were not different than no taping^14^. Based on these pieces of evidence, although taping of patients’ face masks could reduce exhaled air jet toward the periocular area^14,15^, it might have a less clinical importance in terms of bacterial contamination. Moreover, this study and previous data^13^ did not provide significant evidence that patients’ face masks (surgical or cloth masks) were associated with more bacterial dispersion than not wearing any masks. In support of our results, a recent large multicenter retrospective study involving more than 500,000 injections also suggested that universal face mask wearing during intravitreal injection was not associated with the risk of post-injection endophthalmitis^18^. Large scale cohorts would be required to definitely address these issues, although it would be very challenging to conduct given the low incidence of post-injection endophthalmitis.

The most common bacterial strains identified were *Staphylococci* spp. and *Micrococcus luteus.* They were found in similar proportions for both experiment and control agar plates. This was consistent with the fact that staphylococci and micrococci were the most common bacteria found in indoor air environment^19^ and demonstrated the importance of including control agar plates in each of the experiments. Interestingly, Streptococci, the well-known oropharyngeal microbiota associated with severe post-injection endophthalmitis, were identified only in speaking experiment scenarios. This emphasized the importance of keeping silence during an intravitreal injection procedure.

In this study, only particles settled on the agar plates were captured. Active air sampling was not employed and we were not able to detect all particles suspended in the air. However, we believed our approach was reasonable to detect bacteria from oropharyngeal droplets. Further studies with active air sampling would be interesting to corroborate our results. The 2-min exposure time was chosen in each scenario to represent the duration of fenestrated surgical drapes covering the faces in most patients undergoing actual intravitreal injections. This exposure time was also employed in other studies investigating bacterial dispersion using blood agar plates^13,15^.

There were some limitations of this study. The sample size was small, although it was in the same range as most other studies investigating periorbital bacterial dispersion^9,13,14^. Owing to the fact that post-injection endophthalmitis events are rare, bacterial dispersion was chosen as a surrogate outcome but its presence might not necessarily be translated to clinical endophthalmitis. Only tight-fitting surgical masks were investigated since loose-fitting was not a standard practice and would be difficult to control. Facial anatomy and facial hair were not controlled although all subjects were of Thai ethnicity and had no facial hair. The participants in this study were relatively young and healthy and our results might not be generalizable to patients with poor oral hygiene or respiratory infections. Using masks plus surgical drapes could cause the perception of suffocation in old patients with chronic obstructive pulmonary disease. Further studies in older and diseased individuals would be useful. However, our study uniquely focused on the use of fenestrated surgical drapes. Careful study measures including the presence of room control agar plates, internal control agar plates in every scenario, randomization of scenario sequences, washout periods between each scenario, and masking of outcome assessors were employed to minimize study biases as much as possible.

In conclusion, this study suggested that, in general, using fenestrated surgical drapes, either reusable cloth drapes or disposable drapes, did not alter periorbital bacterial growth on blood agar plates. Our results also did not support that patients’ surgical masks were associated with more periorbital bacterial dispersion. Refraining from talking during the intravitreal injection procedures remained the most crucial factor to minimize oropharyngeal bacterial dispersion.

## Data Availability

The datasets generated during and/or analysed during the current study are available from the corresponding author on reasonable request.

## References

[1] Parikh R (2017). Trends of anti-vascular endothelial growth factor use in ophthalmology among privately insured and medicare advantage patients. Ophthalmology.

[2] Storey PP, Patel D, Garg S (2020). Endophthalmitis following intravitreal injection of anti-vascular endothelial growth factor agents. Can. J. Ophthalmol..

[3] Al-Rashaed S, Alsulaiman SM, Alrushood AA, Almasaud J, Arevalo JF (2016). Incidence of endophthalmitis after intravitreal anti-vascular endothelial growth factor: Experience in Saudi Arabia. Middle East Afr. J. Ophthalmol..

[4] Nentwich MM (2014). Endophthalmitis after intravitreal injection: Decreasing incidence and clinical outcome-8-year results from a tertiary ophthalmic referral center. Retina.

[5] Garg S, Dollin M, Hsu J, Storey P, Vander J (2015). Effect of a strict ‘no-talking’ policy during intravitreal injection on post-injection endophthalmitis. Ophthalmic Surg. Lasers Imaging Retina.

[6] McCannel CA (2011). Meta-analysis of endophthalmitis after intravitreal injection of anti-vascular endothelial growth factor agents: Causative organisms and possible prevention strategies. Retina.

[7] Shah CP (2011). Outcomes and risk factors associated with endophthalmitis after intravitreal injection of anti-vascular endothelial growth factor agents. Ophthalmology.

[8] Fileta JB, Scott IU, Flynn HW (2014). Meta-analysis of infectious endophthalmitis after intravitreal injection of anti-vascular endothelial growth factor agents. Ophthalmic Surg. Lasers Imaging Retina.

[9] Wen JC, McCannel CA, Mochon AB, Garner OB (2011). Bacterial dispersal associated with speech in the setting of intravitreous injections. Arch. Ophthalmol..

[10] Doshi RR, Leng T, Fung AE (2012). Reducing oral flora contamination of intravitreal injections with face mask or silence. Retina.

[11] Hadayer A (2020). Patients wearing face masks during intravitreal injections may be at a higher risk of endophthalmitis. Retina.

[12] Dbouk T, Drikakis D (2020). On respiratory droplets and face masks. Phys. Fluids (Woodbury, N.Y.: 1994).

[13] Patel SN (2021). Bacterial dispersion associated with various patient face mask designs during simulated intravitreal injections. Am. J. Ophthalmol..

[14] Schultheis WG (2021). Effect of taping face masks on quantitative particle counts near the eye: Implications for intravitreal injections in the COVID-19 era. Am. J. Ophthalmol..

[15] Raevis JJ (2021). Face masks and bacterial dispersion toward the periocular area. Ophthalmology.

[16] Avery RL (2014). Intravitreal injection technique and monitoring: Updated guidelines of an expert panel. Retina.

[17] Grzybowski A (2018). 2018 update on intravitreal injections: Euretina expert consensus recommendations. Ophthalmologica.

[18] Writing committee for the Post-Injection Endophthalmitis Study Group *et al.* The influence of universal face mask use on endophthalmitis risk after intravitreal anti-vascular endothelial growth factor injections. *Ophthalmology* **128**, 1620–1626. 10.1016/j.ophtha.2021.05.010 (2021). 10.1016/j.ophtha.2021.05.010 PMC8130590 34019955

[19] Nevalainen, A. & Morawaska, L. *Biological Agents in Indoor Environments. Assessment of Health Risks. Work Conducted by a WHO Expert Group between 2000–2003*. (World Health Organization, 2009).

